# 

**Published:** 1875-12

**Authors:** 


Report of Sixteen Cases of Cataract Operations. By B. Joy Jeffries, M.D. Pamphlet; pp. 8.

---

Physiological Action of Lycoctonia. By Isaac Ott, M.D. Pamphlet, pp. 8.

HISTORY OF AMERICAN MEDICAL LITERATURE, from 1776 to the Present Time. By S. D. Gross, M.D., L.L.D., D.C.L. Oxon. Pamphlet, pp. 85.

ARE CARBOLIC ACID DISINFECTANTS USEFUL IN YELLOW FEVER? By Y. R. LeMormier, M.D. Pamphlet; pp. 8.

MANITOU, COLORADO; Its Mineral Waters and Climate. By S. Edwin Sully, M. D. Pamphlet; pp. 40.

CONSOLIDATED REPORT OF CROPS, ETC., Returned to the State Department of Agriculture. By T. P. Janes, Commissioner of Agriculture. Pamphlet; pp. 4.

